# Very high baseline HIV viremia impairs efficacy of non-nucleoside reverse transcriptase inhibitor-based ART: a long-term observation in treatment-naïve patients

**DOI:** 10.1186/s40249-020-00700-8

**Published:** 2020-06-22

**Authors:** Shuai Chen, Yang Han, Xiao-Jing Song, Yan-ling Li, Ting Zhu, Hong-Zhou Lu, Xiao-Ping Tang, Tong Zhang, Min Zhao, Yun He, Sheng-Hua He, Min Wang, Yong-Zhen Li, Shao-Biao Huang, Yong Li, Jing Liu, Wei Cao, Tai-Sheng Li

**Affiliations:** 1 Department of Infectious Diseases, Peking Union Medical College Hospital, Chinese Academy of Medical Sciences, No.1 Shuaifuyuan, Wangfujing Street, Beijing, 100730 China; 2 Department of International Medical Services, Peking Union Medical College Hospital, Chinese Academy of Medical Sciences, No.1 Shuaifuyuan, Wangfujing Street, Beijing, 100730 China; 3 Shanghai Public Health Clinical Center affiliated with Fudan University, Shanghai, China; 4 Guangzhou Eighth People’s Hospital, Guangzhou, China; 5 Beijing You’an Hospital, Capital Medical University, Beijing, China; 6 Fifth Medical Center of Chinese PLA General Hospital, Beijing, China; 7 The Infectious Disease Hospital of Henan Province, Zhengzhou, China; 8 Chengdu Infectious Diseases Hospital, Chengdu, China; 9 The First Hospital of Changsha, Changsha, China; 10 The Center for Disease Prevention and Control of Guangxi province, Nanning, China; 11 Nanning No.4 People’s Hospital, Nanning, China; 12 The Longtan Hospital, Liuzhou, China; 13 The hospital affiliated with the Chinese Medical University, Hangzhou, China

**Keywords:** HIV, Viral load, Baseline RNA, Antiretroviral therapy, Treatment outcome, Virologic response

## Abstract

**Background:**

It is not completely clear whether a very high pre-therapy viral load (≥ 500 000 copies/ml) can impair the virological response. The aim of this study was to examine the influence of very high baseline HIV-RNA levels on long-term virological responses under one type of regimen.

**Methods:**

A retrospective study was performed based on data from two multicenter cohorts in China from January to November 2009, and from May 2013 to December 2015. Untreated HIV infected adults between 18 and 65 years old were recruited before receiving non-nucleoside reverse transcriptase inhibitor-based regimen. All patients had baseline HIV-RNA levels over 500 copies/ml, good adherence, and were followed for at least 24 weeks. Virological suppression was defined as the first HIV-RNA < 50 copies/ml. Virological failure was defined as any of incomplete viral suppression (HIV-RNA ≥ 200 copies/ml without virological suppression within 24 weeks of treatment) and viral rebound (confirmed HIV-RNA level ≥ 50 copies/ml after virological suppression). Chi-square test, Kaplan–Meier analysis, Cox proportional hazards model and Logistic regression were used to compare virological response between each pretreated viral load stratum.

**Results:**

A total of 758 treatment-naïve HIV patients in China were enlisted. Median follow-up time (IQR) was 144 (108–276) weeks. By week 48, rates of virological suppression in three groups (< 100 000, 100 000–500 000 and ≥ 500 000 copies/ml) were 94.1, 85.0, and 63.8%, respectively (*P* < 0.001). Very high baseline HIV viremia over 500 000 copies/ml were found to be associated with delayed virological suppression (≥ 500 000 vs <  100 000, adjusted relative hazard = 0.455, 95% *CI*: 0.32–0.65; *P* < 0.001) as well as incomplete viral suppression (≥ 500 000 vs < 100 000, adjusted odds ratio [a*OR*] = 6.084, 95% *CI*: 2.761–13.407; *P* < 0.001) and viral rebound (≥ 50 000 vs < 100 000, a*OR* = 3.671, 95% *CI*: 1.009–13.355, *P* = 0.048).

**Conclusions:**

Very high levels of pre-treatment HIV-RNA were related with delayed efficacy of NNRTI-based ART and increased risk of treatment failure. More potent initial regimens should be considered for those with this clinical character.

## Background

As the primary treatment for HIV infected patients, the benefits of highly active antiretroviral therapy (ART) in reducing mortalities by suppressing the plasma viral load to an undetectable level has been well established [1–3]. However, poor virological responses to treatment were observed in certain patients, which may require prolonged treatment to achieve the expected virological suppression. There are occasions when patients even fail the primary regimen with absence of own resistance-associated mutations.

Virological response to ART could be related to several factors including age, sex, mode of transmission, drug adherence, drug resistance, hepatitis virus coinfection, and pretreatment HIV-RNA levels [2]. Among the factors that may have an influence on the virological response to ART, the level of baseline HIV-RNA greater than 100 000 copies/ml before the start of treatment was proved an independent predictor of delayed virological suppression as well as increased risk of virological failure, leading to increased mortality in several studies [2, 4–8]. A previous cohort study suggested that a very high pre-treatment viral load greater than 500 000 copies/ml significantly impairs the virological response. This study, however, was limited by diversified choice of regimen, as ritonavir-boosted protease inhibitors (PI/r) was more frequently used in patients with higher baseline viral load due to its high barrier of drug related resistance [9]. Another study confirmed the link between very high baseline viral load and poor virological responses while some crucial factors were ignored, including treatment modifications and adherence [10]. Therefore, the role of a very high baseline HIV-RNA level in long-term virological responses to ART remains unclear, especially with certain types of regimens.

The combination of two nucleoside reverse transcriptase inhibitors (NRTI) + non-nucleoside reverse transcriptase inhibitor (NNRTI) has been recommended as the first-line treatment until 2015, when integrase strand transfer inhibitor (INSTI) based regimen were established [11]. However, NNRTI-based regimens are still widely used as the first-line treatment across middle- and low-income countries including China, for the consideration of accessibility and cost-efficiency [12, 13]. The aim of the present study was to examine the influence of very high baseline HIV-RNA levels on long-term virological responses to the regimen of 2NRTIs + NNRTI in a large cohort of HIV-infected patients.

## Methods

### Participants and study design

A retrospective study was performed based on two completed prospective multi-center cohort in China. Details of both cohorts have been described in detail elsewhere [14, 15]. The data of patients in the two cohorts were collected from January to November 2009, and from May 2013 to December 2015. The cohorts included HIV-treatment naïve patients between 18 and 65 years old. Recruited patients received ART and were followed regularly, when information including HIV-RNA levels, CD4 cell count, adverse effects and adherence were collected. In this study, patients were selected according to the following criteria: 1) available data of baseline HIV-RNA level and CD4 cell count; 2) 2NRTIs + NNRTI as their initial regimen; 3) baseline HIV-RNA over 500 copies/ml; 4) over 98% adherence to the treatment, assessed by self-report, i.e. patients reporting their missed doses from the last time of visit; 5) time of follow-up longer than 24 weeks, to ensure enough time for evaluating efficacy.

Information including age of diagnosis, sex, mode of transmission and clinical characteristics such as HIV subtype, baseline HIV-RNA level, baseline CD4+ T cell count, planned regimen, year of ART initiation and co-infections including seropositivities of hepatitis B virus surface antigen (HBsAg) and hepatitis C virus antibody (HCV-Ab) were collected. Pre-treatment HIV-RNA levels were categorized as < 100 000 copies/ml, 100 000 to 500 000 copies/ml and ≥ 500 000 copies/ml. Baseline CD4 cell count were categorized as < 100 cells/mm^3^, 100–199 cells/mm^3^, 200–350 cells/mm^3^ and >  350 cells/mm^3^. Plasma HIV-RNA level and CD4 cell count at each follow-up after the start of treatment (week 12, 24, 48, 72, 96, 144, 192) were also collected for analysis.

For the available pre-treatment resistance data, we used the Genotypic Resistance Interpretation Algorithm – HIVdb Programme (HIVdb, Stanford University, Stanford, CA) to calculate penalty scores for relevant NRTI and NNRTI, and sequences were determined to be either susceptible (< 15, including potential low-level resistance) or resistant (≥ 15; low-, medium-, or high-resistance).

The prospective cohorts in this study had been approved by Institutional Review Board of Peking Union Medical College Hospital and complied the Principles of Good Clinical Practice and the Declaration of Helsinki. Patients were enrolled in the cohorts after informed consents were signed to provide their anonymized data for academic not-for-profit studies.

### Endpoints

Definitions of virological suppression and virological failure differ between different regions [16, 17]. The present study defined virological suppression as the time point when plasma HIV viral load was less than 50 copies/ml. Virological failure was defined as incomplete viral suppression (defined as HIV-RNA remaining ≥ 200 copies/ml without ever achieving virological suppression by week 24 after the start of the treatment) or viral rebound (defined as confirmed HIV-RNA level ≥ 50 copies/ml after virological suppression). Blips (an isolated HIV-1 RNA at least 50 copies/ml that is immediately preceded and followed by virological suppression) were excluded from patients who met the rebound definition, since it was not related with virological failure according to previous reports [18].

### Statistical analyses

All statistical analyses were performed using the SPSS 24.0 statistical software package (IBM Corporation, Armonk, NY) and GraphPad Prism version 8 (GraphPad Software, Inc., La Jolla, CA). Baseline demographic and clinical characteristics were summarized using medians (interquartile ranges [IQRs]), and frequencies (percentages). Chi-squared test was used in analyzing virological suppression rate in three different groups. Kaplan-Meier curves were used to estimate the time and probability to achieve virological suppression. Multivariable Cox proportional hazard models were used to adjust for potential confounders and outcomes were expressed as ratio hazard (RH) with confidence intervals of 95% confidence interval (95% *CI*). Patients were right-censored if they did not achieve virological suppression but stopped follow-up. Binary Logistic regression was used to evaluate the odds ratio (*OR*) to incomplete viral suppression and viral rebound. Factors with associations with *P* <  0.10 in univariate analysis were entered into the multivariable model. All tests of significance were 2-sided, with a *P* value < 0.05 indicating that an association was statistically significant.

## Results

### Demographic characteristics of the study population

The study sample was based on 758 eligible pre-treatment HIV infected patients. Demographic and clinical characteristics of the included patients are shown in Table 1. Patients were mainly male (565 [74.5%]), with a median age of 33 (24–71) years old. Regarding pre-treatment HIV-RNA level, 27.8 and 6.3% of patients, showed viremia ranging between 100 000–500 000 copies/ml, and ≥ 500 000 copies/ml, respectively. Most selected patients (338 [44.6%]) had baseline CD4 cell count between 200 and 350 cells/mm^3^. The most commonly used regimen was tenofovir disoproxil fumarate (TDF) + lamivudine (3TC) + efavirenz (EFV) (517 [68.2%]). In those whose genetic testing were available, very few had significant resistance to their regimen (16/353 [4.5%]). The median follow-up time of the study population was 144 (108–276) weeks.

**Table 1:** Demographic and clinical characteristics of the study population

Demographic and clinical characteristics	***n*** (%) or median (IQR)
Males	565 (74.5%)
Age (years)	33 (27–41)
Year of ART initiation	
2009	240 (31.7%)
2013–2015	518 (68.3%)
Follow-up time (weeks)	144 (108–276)
Baseline viral load (copies/ml)	
< 100 000	499 (65.8%)
≥ 100 000 and < 500 000	211 (27.8%)
≥ 500 000	48 (6.3%)
Mode of transmission	
Homosexual	290 (38.3%)
Heterosexual	378 (49.9%)
Bisexual	21 (2.8%)
Others^a^	15 (2.0%)
Unknown	54 (7.1%)
Baseline CD4 level (cells/mm^3^)	
< 100	96 (12.7%)
≥ 100 and < 200	143 (18.9%)
≥ 200 and < 350	338 (44.6%)
≥ 350	181 (23.9%)
Subtype	
AE	224 (29.6%)
B/C/BC	111 (14.6%)
Others	17 (2.2%)
Unknown	406 (53.6%)
Regimen	
TDF + 3TC + EFV	517 (68.2%)
3TC + AZT + NVP	78 (10.3%)
3TC + AZT + EFV	14 (1.8%)
3TC + TDF + NVP	7 (0.9%)
Other 2NRTIs + NNRTI	142 (18.7%)
Serum HBsAg status	
Positive	89 (12.1%)
Negative	645 (87.9%)
Serum HCV-Ab status	
Positive	29 (4.0%)
Negative	699 (96.0%)
Resistance	
Low, intermediate and high	16 (4.5%)
Susceptible and potential low	337 (95.5%)

*3TC* Lamivudine, *ART* Antiretroviral therapy, *AZT* Zidovudine, *EFV* Efavirenz, *HBsAg* Surface antigen of the hepatitis B virus, *HCV-Ab* Hepatitis C antibody, *IQR* Interquartile ranges, *NNRTI* Non-nucleoside reverse transcriptase inhibitor, *NRTI* Nucleoside reverse transcriptase inhibitor, *NVP* Nevirapine, *TDF* Tenofovir disoproxil fumarate

^a^Includes blood transfusion, exposure to infected needles, etc.

### Virological trajectories in patients with different levels of baseline HIV-RNA

**Fig. 1:**
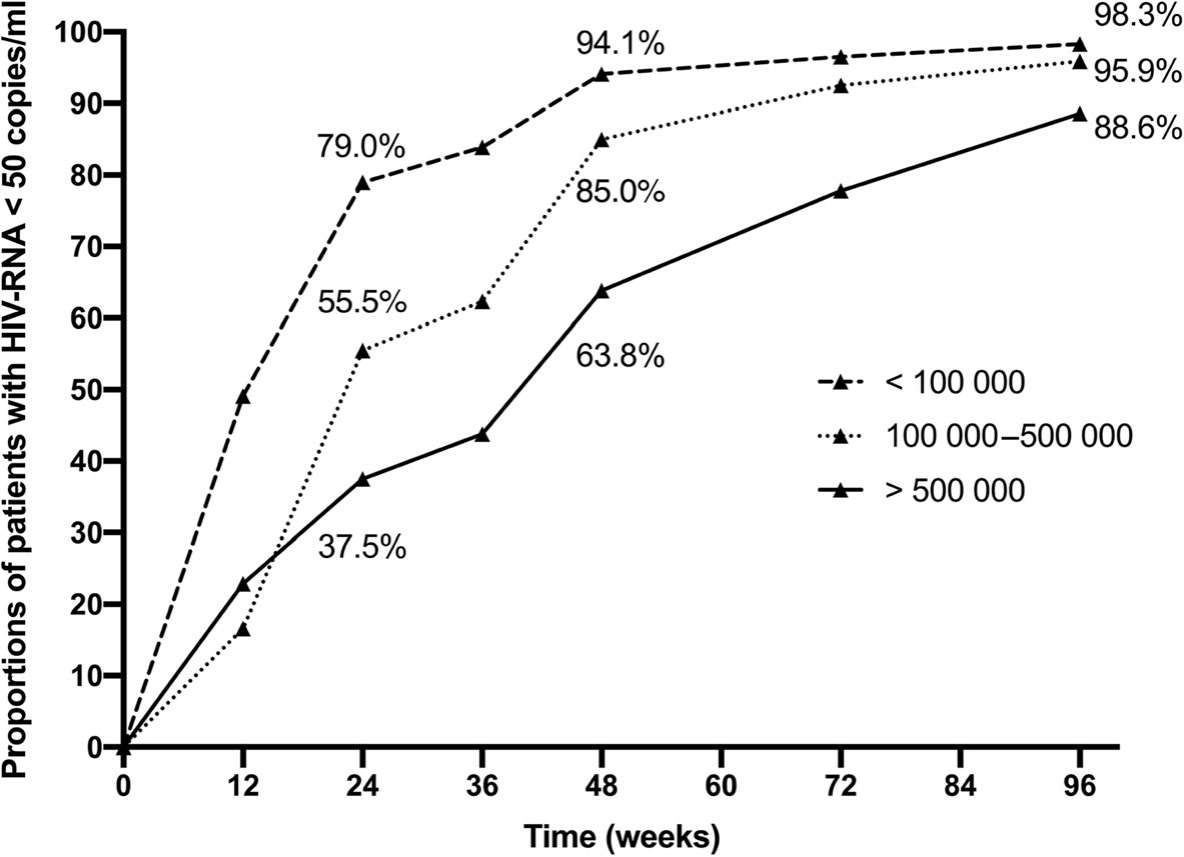
Percentage of patients based on baseline HIV-RNA level who had achieved virological suppression in different follow-up. *P* <  0.001 on Chi-square analysis

**Fig. 2:**
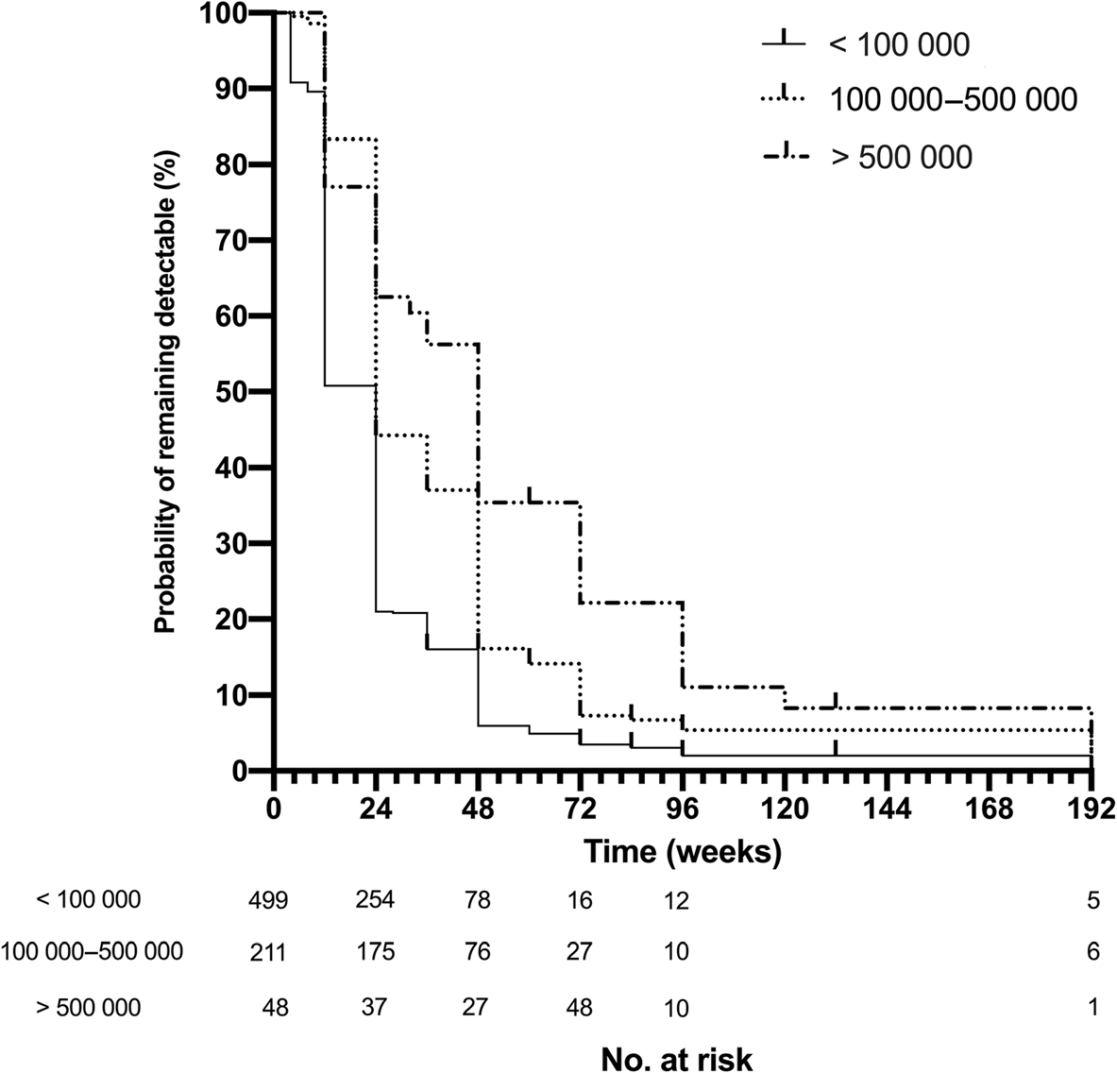
Kaplan–Meier curve of time to virologic suppression based on baseline HIV-RNA levels. Log-rank *P* < 0.001

Overall, 529 (69.8%) of the patients in the cohort had achieved virological suppression by week 24, and 671 (89.7%) patients achieved virological suppression by week 48. At week 24, only 18/48 (37.5%) patients with a baseline HIV-RNA over 500 000 copies/ml had achieved virological suppression, compared with 117/211(55.5%) in those with a baseline between 100 000 and 500 000 copies/ml. Figure 1 shows the percentage of patients that achieved virological suppression in different baseline level groups at week 12, 24, 48, 72 and 96. The rates of virological suppression in those with higher pretreatment viral load remained all the way lower than those with lower pretreatment viral load except for the first 12 weeks. At week 96, 718 (94.7%) patients still remained in the sample cohort, and the percentage of virological suppressed patients in three groups by rising baseline HIV-RNA levels were 88.6, 95.9 and 98.3%, respectively. Chi-square analysis suggested all the difference in percentage between each group at different time points were significant (*P* < 0.001). In addition, the cumulative Kaplan-Meier estimation showed a significantly lower probability of virological suppression for higher HIV-RNA categories (log-rank *P* < 0.001, Fig. 2). After the treatment, the median CD4 cell count of all the patients in the cohort rose from 263 (165–245) cells/m^3^ at week 0 to 446 (319–545) cells/m^3^ at week 96. Table 1S shows the number of patients that remained in three groups and of those who achieved virological suppression in each follow-up.

Univariate and Multivariable Cox proportional hazard models were used to analyze factors associating with time to virological suppression (Table 2). Very high baseline HIV viremia over 500 000 copies/ml were found to be independently associated with delayed virological suppression, after adjusting for factors including sex, age, mode of transmission, HIV-subtype, pre-treatment CD4 cell count, sero-positivity of HBsAg and HCV-Ab (≥ 500 000 vs < 100 000, adjusted RH [aRH] = 0.455, 95% *CI*: 0.319–0.648; *P* <  0.001). Male sex was shown to be another significant relevant factor of deterred virological suppression (male vs female, aRH = 0.755, 95% *CI*: 0.622–0.916, *P* <  0.001). None of age, pre-treatment CD4 cell count, mode of transmission, HIV-subtype, pre-treatment resistance, initial regimen, sero-positivity of HBsAg of HCV-Ab was significantly associated with delayed virological suppression.

**Table 2:** Univariate and multivariate Cox proportional hazard models of the time to virological suppression among 758 treatment-naïve patients initiating NNRTI-based ART. Variables were mutually adjusted in the multivariate model that included age

Variable	Crude			Adjusted		
	RH	(95% ***CI***)	***P***	RH	(95% ***CI***)	***P***
**Male gender**	0.757	(0.641–0.894)	0.001	0.755	(0.622–0.916)	**0.004**
**Year of ART Initiation**	0.981	(0.839–1.148)	0.812	–	–	**–**
**Baseline RNA (copies/ml)**						
< 100 000	1.000	–	–	1.000	–	–
100 000–500 000	0.628	(0.532–0.741)	**< 0.001**	0.602	(0.497–0.730)	**< 0.001**
≥ 500 000	0.477	(0.349–0.652)	**< 0.001**	0.455	(0.319–0.648)	**< 0.001**
**Baseline CD4 (cells/m**^**3**^**)**						
< 100	1.000	–	–	1.000	–	–
100–200	1.057	(0.809–1.383)	0.684	0.970	(0.740–1.272)	0.826
200–350	1.227	(0.970–1.551)	**0.088**	1.046	(0.823–1.331)	0.712
> 350	1.313	(0.016–1.697)	**0.037**	1.181	(0.931–1.532)	0.209
**HBsAg (+)**	0.928	(0.741–1.164)	0.520	–	–	–
**HCV-Ab (+)**	0.878	(0.605–1.275)	0.495	–	–	–
**Mode of transmission**	0.910	(0.722–1.147)	0.	–	–	–
Others	1.000	–	–			
Homosexual	1.093	(0.649–1.839)	0.738	–	–	–
Heterosexual	1.196	(0.713–2.006)	0.498	–	–	–
Bisexual	0.804	(0.408–1.582)	0.527	–	–	–
**Regimen**						
Other 2NRTIs + NNRTI	1.000	–	–	–	–	–
3TC + TDF + EFV	0.963	(0.790–1.175)	0.711	–	–	–
3TC + AZT + NVP	0.923	(0.694–1.227)	0.581	–	–	–
3TC + TDF + NVP	0.669	(0.383–1.167)	0.157	–	–	–
3TC + AZT + EFV	0.742	(0.346–1.590)	0.443	–	–	–
**HIV subtype**	0.991	(0.898–1.093)	0.858	–	–	–
**Resistance (**low, intermediate and high)	0.757	(0.450–1.274)	0.294	–	–	–

*3TC* Lamivudine, *AZT* Zidovudine, *CI* Confidence interval, *EFV* Efavirenz, *HBsAg* Surface antigen of the hepatitis B virus, *HCV-Ab* Hepatitis C antibody, *NNRTI* Non-nucleoside reverse transcriptase inhibitor, *NRTI* Nucleoside reverse transcriptase inhibitor, *NVP* Nevirapine, *TDF* Tenofovir disoproxil fumarate

### High baseline HIV-RNA is related to virological failure

In the sample cohort, 71/758 (9.4%) patients had incomplete viral suppression, among whom 21/71 (30.0%) had never achieved virological suppression. 23/758 (3.0%) patients had viral rebound, with 7/23 (30.4%) having additional blips, 1/23 (4.3%) having severe adverse effect and stopped the initial regimen in week 48. Of these patients, 3/94 (3.2%) were resistant to their initial regimen that was later proved by genetic analysis.

Binary Logistic regression models were built to evaluate factors related to incomplete viral suppression or viral rebound (Tables 3 and 4). After adjusting with covariates including age, sex, HIV subtype, pre-treatment resistance, baseline CD4 level, sero-positivity of HBsAg or HCV-Ab, mode of transmission, odds ratio (*OR*) to incomplete viral suppression were significantly higher in patients with baseline HIV-RNA levels ≥ 500 000 copies/ml (≥ 500 000 vs < 100 000, a*OR* = 6.084, 95% *CI*: 2.761–13.407; *P* < 0.001). After excluding patients with viral blips, baseline HIV viremia over 500 000 copies/ml were found to be independently associated with rebound (≥ 500 000 vs < 100 000, a*OR* = 3.671, 95% *CI*: 1.009–13.355; *P* = 0.048).

**Table 3:** Univariate and adjusted Logistic regression analysis of *OR* of incomplete suppression among 758 treatment-naïve patients initiating NNRTI-based ART

Variable	Univariate analysis			Adjusted analysis †		
	***OR***	(95% ***CI***)	***P*** value	***OR***	(95% ***CI***)	***P*** value
**Male gender**	1.608	(0.861–3.004)	0.136	1.533	(0.805–2.917)	0.193
**Age**	0.993	(0.969–1.018)	0.595	–	–	**–**
**Year of ART initiation**	0.743	(0.448–1.230)	0.248	–	–	**–**
**Baseline RNA (copies/ml)**						
< 100 000	1.000	–	–	1.000	–	–
100 000–500 000	2.937	(1.712–5.041)	**< 0.001**	2.736	(1.577–4.747)	**< 0.001**
≥ 500 000	6.458	(3.061–13.625)	**< 0.001**	6.084	(2.761–13.407)	**< 0.001**
**Baseline CD4 (cells/m**^**3**^**)**						
< 100	1.000	–	–	1.000	–	–
	1.057	(0.809–1.383)	**0.014**	1.386	(0.633–3.307)	0.414
200–350	1.227	(0.970–1.551)	**0.004**	0.970	(0.457–2.059)	0.938
> 350	1.313	(0.016–1.697)	0.120	0.478	(0.190–1.206)	0.118
**HBsAg (+)**	1.246	(0.612–2.536)	0.545	–	–	–
**HCV-Ab (+)**	1.197	(0.350–4.093)	0.774	–	–	–
**Mode of transmission**	1.029	(0.734–1.444)	0.866	–	–	–
**HIV subtype**	1.306	(0.631–2.705)	0.472	–	–	–
**Resistance (**low, intermediate and high)	1.543	(0.328–7.261)	0.583	–	–	–

*CI* Confidence interval, *HBsAg* Surface antigen of the hepatitis B virus, *HCV-Ab* Hepatitis C antibody

†Significance in Omnibus Test of Model Coefficients is < 0.001. Significance in Hosmer and Lemeshow Test is 0.498

**Table 4:** Univariate and adjusted Logistic regression analysis of *OR* of viral rebound after excluding blips among 758 treatment-naïve patients initiating NNRTI-based ART

Variable	Univariate analysis			Adjusted analysis ^**a**^		
	***OR***	(95% ***CI***)	***P*** value	***OR***	(95% ***CI***)	***P*** value
**Male gender**	1.259	(0.461–3.440)	0.653	–	–	–
**Age**	0.976	(0.932–1.021)	0.286	–	–	**–**
**Year of ART initiation**	0.551	(0.267–1.137)	0.107	–	–	**–**
**Baseline RNA (copies/ml)**						
< 100 000	1.000	–	–	1.000	–	–
100 000–500 000	2.781	(1.113–6.950)	**0.029**	2.405	(0.943–6.133)	0.066
≥500 000	5.182	(1.592–17.555)	**0.008**	3.671	(1.009–13.355)	**0.048**
**Baseline CD4 (cells/m**^**3**^**)**						
< 100	1.000	–	–	1.000	–	–
100–200	0.781	(0.254–2.402)	0.666	0.936	(0.297–0.946)	0.910
200–350	0.318	(0.104–0.971)	**0.044**	0.469	(0.144–1.524)	0.208
> 350	0.250	(0.061–1.024)	**0.054**	0.350	(0.082–1.494)	0.156
**HBsAg (+)**	2.049	(0.071–5.668)	0.167	–	–	–
**HCV-Ab (+)**	1.060	(0.138–8.148)	0.955	–	–	–
**Mode of transmission**	1.054	(0.594–1.871)	0.858	–	–	–
**HIV subtype**	3.168	(0.777–12.922)	0.108	–	–	–
**Resistance (**low, intermediate and high)	1.747	(0.210–14.501)	0.605	–	–	–

*CI* Confidence interval, *HBsAg* Surface antigen of the hepatitis B virus, *HCV-Ab* Hepatitis C antibody

^a^Significance in Omnibus Test of Model Coefficients is 0.037. Significance in Hosmer and Lemeshow Test is 0.788

## Discussion

As reported by many previous studies, including some large-scale studies with over 100 000 participants, that baseline HIV viral load over 100 000 copies/ml is associated with longer time to virological suppression as well as increased incidence of viral rebound [6–8], yet very few had addressed the impact of baseline viral load over 500 000 copies to virological effect of ART [9, 10]. The present study reports virological changes of 758 treatment-naive HIV-infected patients from two prospective cohort in long-term follow-up. Our study demonstrates that patients with very high baseline HIV-RNA levels, particularly those of over 500 000 copies/ml, were significantly associated with delayed or incomplete virological suppression and increased occurrence of viral rebound on NNRTI-based regimens. In addition, the declining RH of virological response following viral load stratum rising suggested a decreased response with increased baseline viral load may constitute a continuum. Our findings from prospective cohorts, in combination with previous studies, further emphasized the influence of baseline HIV-RNA > 500 000 copies/ml on virological response based on NNRTI regimens.

INSTI-based ART, in specific dolutegravir (DTG)- or raltegravir (RAL)-based regimens have been recommended as the first-line regimen in most developed countries [16, 17] for their higher efficacy, less adverse effects and better tolerance [19]. However, clinical and programmatic experience with INSTI in low- and middle-income countries is limited due to the relatively high cost [20]. Currently, NNRTI-based ART regimens such as TDF + 3TC + EFV are still recommended as alternative first-line regimens according to WHO guidelines [12] and are still commonly used in many developing countries [21]. In addition, DTG-based ART may not be as cost-effective as EFV according to studies in many countries [22, 23], presenting other problems if completely replacing the latter very soon. On the other hand, there has been some studies demonstrating that the virological efficacy of EFV is better than NVP, as both-based regimen were used in this study [24]. We made additional comparison between the number of patients being treated with EFV or NVP based regimen and the proportion in their baseline viral load strata, and it was shown that there was no predilection of either drug in any viral load stratum (Chi-square test, *P* = 0.896).

Recent guidelines have recommended starting ART as soon as HIV infection is confirmed, regardless of HIV-RNA level or time of infection so as to get rapid suppression of viral load [16]. In the present study, only 37.5% of the patients with baseline HIV-RNA ≥ 500 000 copies/ml achieved virologic suppression at week 24, much less than those with lower baseline HIV-RNA levels. This trend continued into 96 weeks of treatment, though by week 96 the differences of virologically suppressed percentage in all three viral load strata were getting smaller, suggesting that most patients may achieve virological suppression given long-term treatment. In this study, in patients who met the definition of incomplete suppression, only 21 never achieve virological suppression during their follow-ups, while the rest of them had their viral load suppressed to undetectable in more than 24 weeks. However, it should be noted that all patients meeting the definition of incomplete suppression need to be evaluated as virological failure, as delayed or incomplete virological suppression will increase risks of opportunistic infections and secondary transmission, and some of these patients may never achieve virological suppression under the current regimen. In addition, we found that high baseline HIV viral load was associated with increased risk of viral rebound, as is consistent with previous studies [9]. The difficulty of NNRTI-based regimens in suppressing such high viral loads indicated that more potent initial regimens, such as INSTI-based regimen should be preferably considered for these patients for early and rapid suppression of viral loads.

In the present study, male sex was found to be an independent risk factor of delayed virologic suppression in multivariate Cox proportional hazard analysis but not significantly related to incomplete suppression or viral rebound. We made an additional comparison of proportions of male and female patients in each viral load stratum, finding that there was no difference in distribution of both sexes (Chi-square test, *P* = 0.136). In sub-analysis within each HIV-RNA group, male sex was still significantly related with postponed virological suppression (*P* = 0.004 for HIV-RNA > 100 000 copies/ml, and *P* = 0.011 for HIV-RNA < 100 000 copies/ml). The association between male sex and postponed virological suppression in this study is consistent with the result of one previous study [10]. Some previous analyses showed similar antiretroviral efficacy in men and women [25–28], in terms of the percentage of virological suppression instead of survival time. Sex was not significantly relevant to incomplete suppression or viral rebound in our study either, but this may be due to limited number of cases. There are differences in pharmacokinetics and pharmacodynamics between sexes due to differences in body composition, sex hormones, microbiome, genetic and immunological differences [29, 30], and the response to treatment as measured by CD4+ T cell count recovery has been reported to favor women [31]. As HIV-infected women enrollment were limited by the major route of transmission in many trials and observations [28] including this study, the role of sex in viral response needs to be further studied.

Positive HBsAg and HCV-Ab were not significantly relevant with virological response in the present study. Similar conclusions have been reached by previous studies [32–34], yet some recent studies suggested that positive HBcAb or HCV-Ab may have adverse effects on the virological response [35, 36]. However, all studies demonstrated that both hepatitis virus seropositivities can decrease the recovery rate of CD4 T cells [32, 34–36], and this was not referred in the present study.

The present study has some limitations. Firstly, the 95% *CI* in analysis of high baseline HIV-RNA and virological failure was wide due to a relatively small number of failed cases, as well as very high baseline HIV-RNA level (≥ 500 000 copies/ml) patients. In this analysis, however, the *OR* of virological failure increased as we raised the viral load stratum. The result would be more convincing if more cases were included. Secondly, adherence in this study was assessed by self-report, which may be less accurate than other methods such as pharmacy refilling, electronic adherence monitoring device [37]. As the adherence may be overestimated here, this might also have contributed to the delayed or incomplete virological response in these patients. Thirdly, profiles of pre-treatment resistance and HIV subtype were not available for all patients in our study. The rate of primary drug resistance in China was around 3.0–4.32% [38]. We analyzed drug resistance in virologically failed patients and found transmitted drug resistance was not main contributor for their treatment failure. For those who had never achieved virological suppression, there are several factors that can be attributed to: unreported inadherence, unsatisfying gastrointestinal absorption, drug-drug interactions, and acquired drug resistance [39], yet the exact reason of failed virological suppression in these patients were not able to be ascertained in this study.

## Conclusions

Our findings in this study support that very high pre-treatment HIV-RNA levels are associated with impaired virological response. Male sex was also found to be related to postponed virological undetectability. Patients with very high HIV-RNA levels, especially those with baseline RNA over 500 000 copies/ml may possibly need some potent regimens as their initial treatment.

## Supplementary information

**Additional file 1: Table 1S.** Number of patients that achieved virological suppression and number of patients that remained in the cohort in each follow-up checkpoint.

## Data Availability

Datasets used in this analysis are available from the corresponding author upon request.

## Abbreviations

3TC: Lamivudine
AZT: Zidovudine
DTG: Dolutegravir
EFV: Efavirenz
ART: Antiretroviral therapy
HIV: Human immunodeficiency viruses
INSTI: Integrase strand transfer inhibitor
NVP: Nevirapine
NRTI: Nucleoside reverse transcriptase inhibitor
NNRTI: Non-nucleoside reverse transcriptase inhibitor
PI: Protease inhibitor
RH: Relative hazard
TDF: Tenofovir disoproxil fumarate
*OR*: Odds ratio
VS: Virological suppression
WHO: World Health Organization
