# Supplementary material for: Very high baseline HIV viremia impairs efficacy of non-nucleoside reverse transcriptase inhibitor-based ART: a long-term observation in treatment-naïve patients
Source: Infect Dis Poverty. 2020 Jun 22;9:75. doi: 10.1186/s40249-020-00700-8 (PMC7310120; doi:10.1186/s40249-020-00700-8)
Supplement: Supplementary file 1 — Additional file 1: Table 1S. Number of patients that achieved virological suppression and number of patients that remained in the cohort in each follow-up checkpoint. [file 40249_2020_700_MOESM1_ESM.docx]

**Table 1S** Number of patients that achieved virological suppression and number of patients that remained in the cohort in each follow-up checkpoint

|  | **No. of patients that achieved virological suppression/No. of patients that remained in the cohort (%) in different follow-up** | | | | | | |
| --- | --- | --- | --- | --- | --- | --- | --- |
| **HIV-RNA stratum** | **week 0** | **week 12** | **week 24** | **week 36** | **week 48** | **week 72** | **week 96** |
| < 100 000 | 0/499 (0%) | 245/499 (49.1%) | 394/499 (79.0%) | 417/497 (83.9%) | 466/495 (94.1%) | 475/492 (96.5%) | 471/479 (98.3%) |
| 100 000–500 000 | 0/211 (0%) | 35/211 (16.6%) | 117/211 (55.5%) | 129/207 (62.3%) | 175/206 (85.0%) | 185/200 (92.5%) | 187/195 (95.9%) |
| ≥ 500 000 | 0/48 (0%) | 11/48 (22.9%) | 18/48 (37.5%) | 21/48 (43.8%) | 30/47 (63.8%) | 35/45 (77.8%) | 39/44 (88.6%) |
| Total | 0/758 (0%) | 291/758 (38.4%) | 529/758 (69.8%) | 567/752 (75.4%) | 671/748 (89.7%) | 695/737 (94.3%) | 697/718 (97.1%) |
